# Breeding Sites of *Phlebotomus sergenti*, the Sand Fly Vector of Cutaneous Leishmaniasis in the Judean Desert

**DOI:** 10.1371/journal.pntd.0001725

**Published:** 2012-07-03

**Authors:** Aviad Moncaz, Roy Faiman, Oscar Kirstein, Alon Warburg

**Affiliations:** Department of Molecular Genetics and Microbiology, The Institute for Medical Research Israel-Canada, The Kuvin Centre for the Study of Infectious and Tropical Diseases, The Hebrew University - Hadassah Medical School, The Hebrew University of Jerusalem, Jerusalem, Israel; National Institutes of Health, United States of America

## Abstract

Phlebotomine sand flies transmit *Leishmania*, phlebo-viruses and *Bartonella* to humans. A prominent gap in our knowledge of sand fly biology remains the ecology of their immature stages. Sand flies, unlike mosquitoes do not breed in water and only small numbers of larvae have been recovered from diverse habitats that provide stable temperatures, high humidity and decaying organic matter. We describe studies designed to identify and characterize sand fly breeding habitats in a Judean Desert focus of cutaneous leishmaniasis. To detect breeding habitats we constructed emergence traps comprising sand fly-proof netting covering defined areas or cave openings. Large size horizontal sticky traps within the confined spaces were used to trap the sand flies. Newly eclosed male sand flies were identified based on their un-rotated genitalia. Cumulative results show that *Phlebotomus sergenti* the vector of *Leishmania tropica* rests and breeds inside caves that are also home to rock hyraxes (the reservoir hosts of *L. tropica*) and several rodent species. Emerging sand flies were also trapped outside covered caves, probably arriving from other caves or from smaller, concealed cracks in the rocky ledges close by. Man-made support walls constructed with large boulders were also identified as breeding habitats for *Ph. sergenti* albeit less important than caves. Soil samples obtained from caves and burrows were rich in organic matter and salt content. In this study we developed and put into practice a generalized experimental scheme for identifying sand fly breeding habitats and for assessing the quantities of flies that emerge from them. An improved understanding of sand fly larval ecology should facilitate the implementation of effective control strategies of sand fly vectors of *Leishmania*.

## Author Summary

Sand flies are small blood sucking flies that transmit *Leishmania*, the etiologic agent of leishmaniasis - a prevalent disease over large areas of the World. Unlike mosquitoes, sand flies do not breed in water. Their larvae develop in humid habitats containing decaying organic matter (e.g. habitats such as burrows, tree holes and caves). However, in most cases, larval breeding habitats are unknown and larvae remain inaccessible to control efforts. In this paper we identified the breeding sites of an important sand fly vector of cutaneous leishmaniasis by using emergence traps to collect adult sand flies exiting caves and cracks. We identified young male sand flies (less than 24 hours old) by examining their external sex organs. The data collected enabled us to determine that sand flies were breeding primarily inside caves and in adjacent cracks but also in man-made support walls constructed with large boulders. These findings will be useful for applying more effective sand fly and leishmaniasis control measures.

## Introduction

The leishmaniases are a group of diseases endangering some 350 million people in 88 countries, most of them in the poorer regions of the globe. The two major clinical forms are cutaneous leishmaniasis (CL) and visceral leishmaniasis (VL). CL manifests as a sore at the bite site of the infected sand fly and is usually self healing. VL is a life-threatening systemic infection. There are an estimated 1–1.5 million cases of CL and half a million new cases of VL annually [1], [2]. CL caused by *Leishmania tropica* and *L. major* are considered emerging diseases in Israel as well as other East Mediterranean countries [3], [4].

The vectors of leishmaniasis are blood-sucking phlebotomine sand flies (Diptera: Psychodidae) belonging to two genera, *Phlebotomus* in the Old World, and *Lutzomyia* in the New World. There are some 700 known species of sand flies but only about 30 of those transmit leishmaniasis to humans [5], [6]. Sand flies are small and fragile nocturnal insects that normally fly close to the ground and refrain from flight activity under windy conditions. Although experimentally marked flies have occasionally been demonstrated to travel over a kilometer, most sand flies remain within several hundred meters of their breeding place during their entire life [6]. Because of their limited flight range, transmission of *Leishmania* within CL endemic areas is often geographically discontinuous, with characteristically small and separate foci close to the reservoir host habitats [7], [8].

The widest gap in our understanding of sand fly biology remains their larval ecology. Sand flies, unlike mosquitoes, do not breed in water and there is relatively little information on their breeding sites [9]. Small numbers of larvae have been recovered from diverse habitats including caves, crevices, animal burrows, termite mounds, cracks in the soil, domestic animal shelters, cracked walls, tree-holes, birds' nests and leaf litter [9], [10]. However, there are only two documented examples of more productive sites: one from Sardinia, where several hundred *Ph.* (*Larrousius*) spp. larvae were recovered from top soil inside an abandoned shed [11], [12], [13] and another from Panama where over two thousand *Lutzomyia* spp larvae were found in soil samples obtained from forest floors [14].

In the insectary, optimal rearing conditions for different sand fly species are often remarkably uniform. For example, desert dwelling *Ph. papatasi* from the Middle East and Neo-tropical *Lu. longipalpis* from Latin America, are optimally reared under the same conditions (26±2°C, 85–95% RH, composted rabbit feces-based larval diet) [15], [16], [17]. Such observations coupled with scanty field studies, indicate that in nature, immature sand flies develop in moist and dark microhabitats affording stable climatic conditions. Eggs are deposited separately and hatch within 7–10 days. Larvae feed upon composted organic matter of animal and plant origin and undergo four larval instars lasting around three weeks. Pupal development lasts 7–10 days [6].

Kfar Adumim is a small township in the Judean Desert where CL caused by *L. tropica* was initially documented in the early 1990s [18]. A more thorough ecological study performed some 10 years later, characterized the *L. tropica* strains from patients and *Ph. sergenti* sand flies [19]. Sporadic cases of CL have continuously been reported from the area since then and sand fly populations, have been monitored intensively [20]. *Ph. sergenti* were shown to be primarily exophilic but towards the end of summer their numbers indoors increased [21]. On the other hand the majority of sand flies captured inside houses were *Ph. papatasi* but only few were collected outside [19], [21]. Significantly, despite the existence of its known vector, *Ph. papatasi*, CL caused by *L. major* is absent from the region probably because the reservoir hosts *Psammomys obesus* are not found in rocky terrain [4], [19].

The current study was designed to characterize larval breeding habitats in an arid region that supports exceptionally dense sand fly populations comprising chiefly one species, *Ph. sergenti* [20], [21], [22]. We were specifically interested to determine whether *Ph. sergenti* breed only in natural habitats or if they may adapt to man-made habitats such as gaps between boulders forming artificial support walls of irrigated gardens. Characterization of breeding sites of *Ph. sergenti* may facilitate the application of larval source-reduction as a component within integrated sand fly control strategies [23].

## Methods

### Study area

Kfar Adumim (31°49′N : 35°20′E) is a rural community located 20 km east of Jerusalem (altitude 316 m). Climate is semiarid with 260 mm mean annual rainfall, and 20°C mean annual temperature. Flora is predominated by perennial desert shrubs and annual grasses [24]. The study area was located on the lime-stone slopes to the south east of the village and in the gorge below. Parts of the slope were strewn with large rocks and debris left over from the construction of the houses and the road above. The slope itself comprises alternate strata of hard flint and soft chalk producing natural terraces, perforated with small caves and cervices. The crevices were occupied by rodents such as spiny mice (*Acomys cahirinus*) and the larger caves were frequently used by rock hyraxes (*Procavia capensis*), the principal reservoir hosts of *L. tropica* in Israel [4], [8], [25].

**Figure 1:**
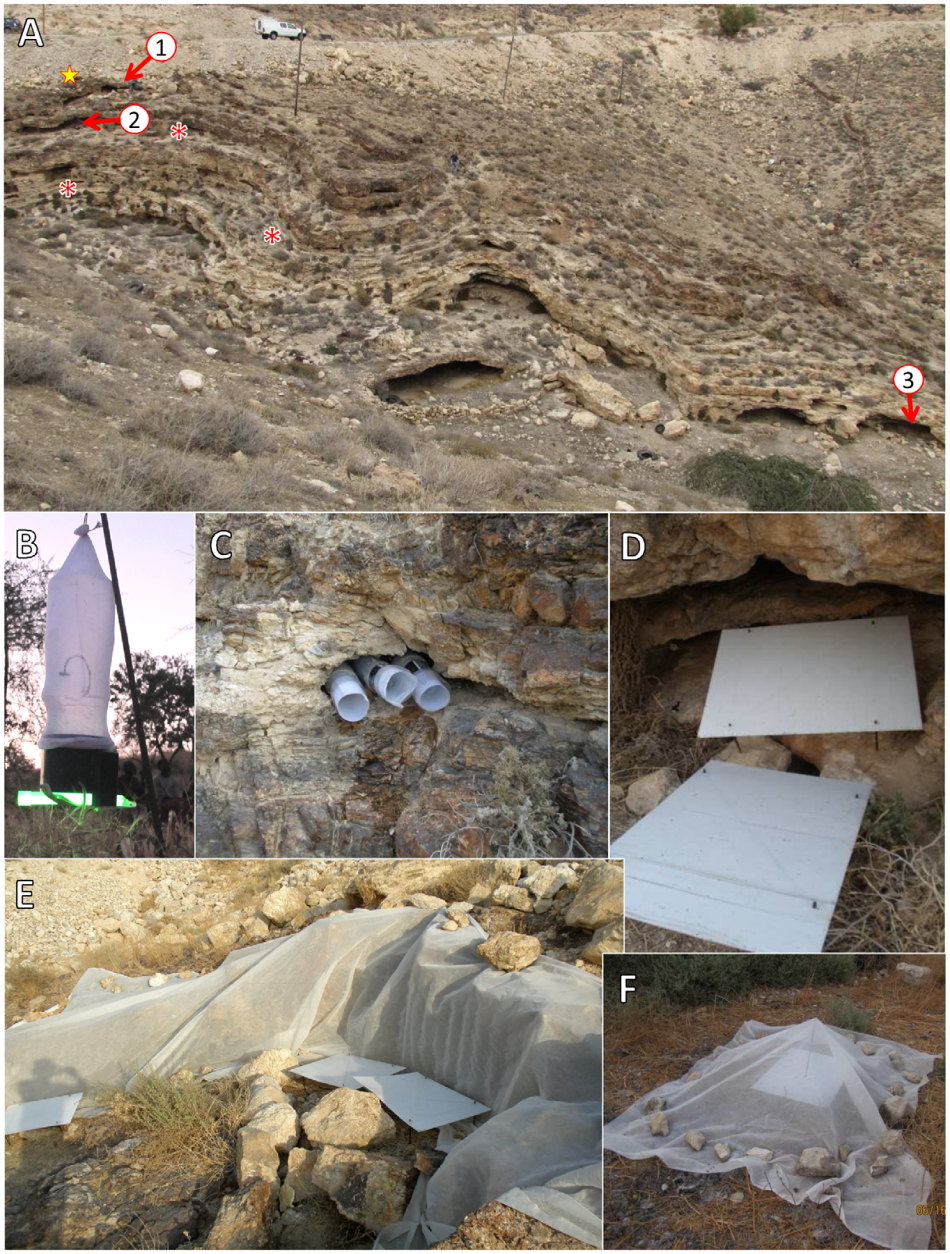
Study sites and trapping methods. **A**: General view of the main study area on the outskirts of Kfar Adumim showing the three cave systems (marked 1,2, & 3 - photo taken facing north), rock slide resulting from the excavation of the road above (marked with yellow star) and rocky ledges with numerous small openings and cracks (red asterisks). **B**: Modified CDC light trap deployed updraft and baited with green light-stick. **C**: A4 sticky traps rolled up in rock crevices to trap emerging sand flies. **D**: Large (60×80 cm) sticky traps deployed horizontally on metal frames. **E**: Cave system No. 1 covered with sand fly-proof mesh to assess emergence of sand flies. Large sticky traps were deployed both inside and outside the cave(s) **F**: Tent-type emergence trap covering an area of approximately 2 m^2^ with single large sticky trap (60×80 cm) inside a sand fly proof net suspended over a central pole.

Three cave systems were explored during the summers of 2010 and 2011:

1. Cave No 1 located 3.5 m below a paved road on a 3–5 m wide rocky ledge, was closest to the village. The system comprised three caves and two smaller caverns that opened into a common anteroom (160 cm wide×66 cm high). An additional cavern (opening 50×140 cm) was located along the same ledge about 5 m from the main complex (marked 1 in Figure 1A).
2. Cave No 2 (680 cm wide×75 cm high) was located on a lower ledge 2 m below cave system No 1 (marked 2 in Figure 1A).
3. Cave system No 3 located at the bottom of the slope near the dry river bed, comprised a large cave (500 cm wide×120 cm high×250 cm deep) with a sandy bottom. A shallow chamber (530 cm wide×140 cm high×180 cm deep) was connected to the main cave via a 400 cm long tunnel (marked 3 in Figure 1A).

**Figure 2:**
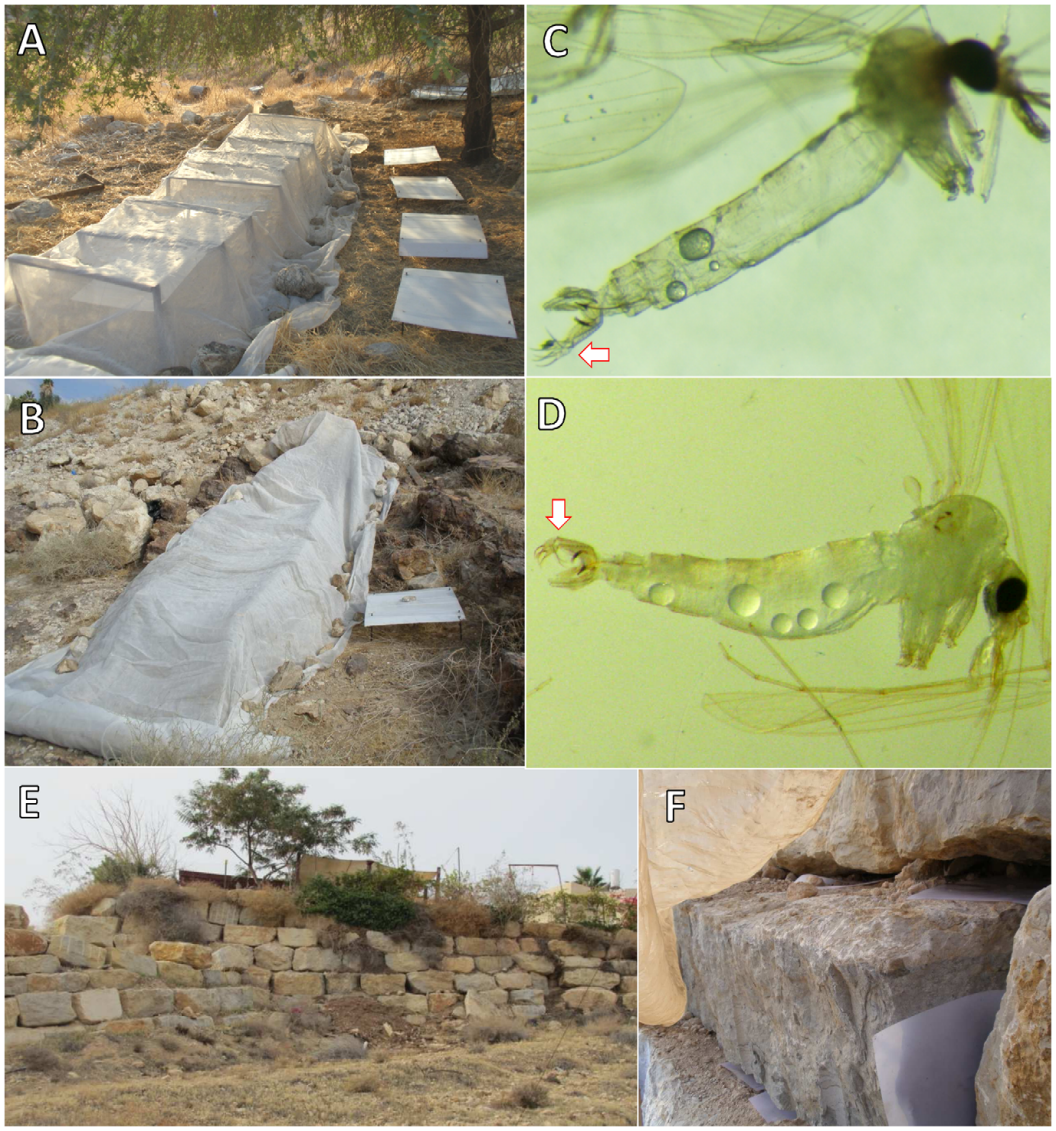
Study sites and trapping methods (contd.). **A**: Tunnel emergence trap comprising four large horizontal sticky traps covered with sand fly-proof netting. Four uncovered sticky traps are included for control. **B**: Tunnel emergence trap placed on a slope strewn with loose rocks. One uncovered trap placed for control. **C**: immature (juvenile) *Phlebotomus sergenti* male with un-rotated external genitalia. Note the ventral orientation of the style (arrow). **D**: A mature *Phlebotomus sergenti* male with fully rotated external genitalia. Note the dorsal orientation of the style (arrow). **E**: Support wall below a house with an irrigated garden. **F**, A4 sticky traps inserted between the boulders of a support wall that was covered with a sand-fly proof mesh.

Other putative breeding and/or resting habitats studied included: natural rocky ledges (some marked by red asterisks) with abundant nooks and cervices, dry river beds, shady areas under trees close to river beds. Artificial habitats were also investigated. These included rock piles (marked with yellow star in Figure 1A) as well as support walls constructed down-slope from houses and gardens. These walls were made of layers of very large boulders placed one on top of the other leaving 2–5 cm gaps. Somewhat wider gaps of 15–20 cm were left between adjacent boulders in the same tier (Figs 2E, 2F). The gardens and lawns above the support wall were irrigated regularly.

### Trapping methods

#### Modified CDC light trap

Powered by two 1.5 V batteries and baited with a green chemical-light sticks found effective for attracting sand flies (Moncaz et al. unpublished)(Cyalume Technologies, Inc. West Springfield, MA, USA), these traps were positioned in updraft orientation with the opening 10–15 cm above ground level (Fig. 1B) [26]. CDC light traps were deployed in and around caves to assess the sand fly population densities in and around potential breeding and/or resting habitats.

#### Standard sticky traps

A4 size printing papers smeared with castor oil, and were inserted into small crevices, rock cracks and gaps between boulders either rolled up (Fig. 1C) or left flat (Fig. 2F).

#### Large sticky traps

A white polypropylene board, measuring 60×80 cm, was placed horizontally on a square metal frame supporting it approximately 15 cm above ground (Fig. 1D). Only the top sides of the boards were smeared with castor oil because prior studies had shown that hardly any flies adhered to the bottom of sticky traps (Moncaz & Warburg, unpublished). Large sticky traps were used independently to monitor sand fly populations (Figs. 1D, 1E, 2A, 2B,) or, when covered by mesh, as an integral part of emergence traps (1F,2A).

#### Emergence trap

Emergence traps for soil, cracks, riverbeds on relatively homogenous flat or sloping terrain comprised a large sticky trap covered with a sand-fly proof net suspended over a central pole. Such traps covered an area of approximately 2 m^2^ (Fig. 1F).

“Tunnel” emergence traps to cover large areas exceeding 10 m^2^, were constructed in the shape of a tunnel. These traps comprised sand fly proof nets (190 holes per square centimeter) that were suspended over metal or wooden frames to enclose an area 6–8 m long by 1.5 m wide. Several sticky traps were placed inside. Tunnel emergence traps were deployed for 1–4 nights (Fig. 2A,B).

#### Emergence traps for caves

Several large sticky traps were placed inside the caves. The cave openings were covered with a sand fly proof net (190 holes per square centimeter), to prevent entry and exit of sand flies from caves (Fig. 1E). The surrounding areas were scanned and alternative exits (if any) were sealed. Additional sticky traps were placed outside the net. Flies were collected and traps were smeared with a fresh coat of oil, daily.

#### Emergence traps for use on support walls

Three 10 m long by 3 m high sections of a support wall were covered with a sand fly-proof net. Standard A4 paper sticky traps were placed in gaps between boulders (Fig. 2E, 2F). Sand flies were collected in the mornings, sticky traps were replaced daily and the nets were rearranged to cover the wall. Sand flies were collected on five consecutive nights.

### General procedures

Sand flies were removed from the sticky straps using fine watchmakers' forceps and placed in ethanol. Traps were wiped clean and smeared again with castor oil. Emergence studies were conducted over consecutive nights in order to distinguish between resting and emerging sand flies. Those exiting during the first night were considered either resting or emerging sand flies. On the other hand, those flies captured 24 hours and longer after the cave (or other habitat) had been covered, were considered more likely to be flies emerging from breeding sites [27].

### Sand flies

In the laboratory, sand flies were placed in a strainer and washed with dilute detergent solution to remove oil and other debris. For identification, sand flies were mounted in Hoyer's medium with their heads separate from thoraces. Flies were identified to species based on cibarial and pharyngeal armature as well as spermathecae of females and external genitalia of males [28], [29], [30]. For all other purposes, flies were kept in 70% ethanol.

### Age-grading of wild-caught male sand flies

The external genitalia of male sand flies rotate on the longitudinal body axis through 180° during the initial 16–24 hours of adult life to assume their mature ( = rotated) position (see experimental data below). Therefore, males with un-rotated or partially rotated external genitalia can be considered to have been captured during their first night of activity as adults.

### Timing the rotation of male genitalia

Like other dipterans, male phlebotomines eclose from the pupae with un-rotated genitalia (Fig. 2C) [31], [32]. In order to make use of this easily discernable physical characteristic to identify young males, we needed to establish the timing of the rotation of male genitalia. *Ph. sergenti* adults were collected in the study area using CDC light traps and colonized using standard methods [16]. Emerging F1 male sand flies were removed from the breeding pots at intervals of 5 hours and placed in the freezer. Thereafter, these male flies were mounted in Hoyer's medium on microscope slides and the position of their genitalia was determined under a microscope at ×100–200 (Figs. 2C, D).

### Soil samples

Ten soil samples were collected in and around caves 1–3 and several sites in the dry riverbed below (Fig. 1A) as well as from cracks in an artificial support wall (Fig. 2E). Selection of sites to be sampled was conducted after the sand fly data had been analyzed in order to provide a well balanced representation of the ecosystems under study. There was no possibility of reaching the depths of caves and gaps between boulders in order to sample the actual breeding site of the larvae. Thus, samples comprising top soil, were weighed in the field, sifted over 2 mm sieve and sealed in heavy plastic sample bags for transport.

In the laboratory a 2.0 g aliquots were removed from each sample, dried in an oven at 105°C for 24 hrs and weighed again. The hygroscopic water content was calculated as the ratio of weight loss to dry weight [33].

To determine the pH, electrical conductivity and salinity, equal weights of air-dried soil and deionized water (30 g) were mixed and allowed to equilibrate for one hour. The mixture was shaken well using a rotary shaker (135 rpm for 5 min), and centrifuged (8,000 rpm for 10 min at 25°C). The supernatant was decanted; pH was measured using a pH meter model SA 520 (Orion Research Inc., Beverly, MA, USA,). Electrical conductivity was determined using a TH-2400 conductometer (El-Hamma Instruments, Mevo-Hamma, Israel) and the salinity was derived from the conductivity values.

To determine values for organic matter, soil aliquots weighing 3 g each (3 aliquots per sample) were subjected to dry combustion (450°C, 8 hr) and reweighed. The weight of combustible organic matter was calculated after reducing the gravimetric water content.

The soil texture was established based on particle sedimentation rates using the hydrometer method [34].

### Statistical methods

The numbers of sand flies captured on the first and second nights by traps placed inside and outside sealed caves were tested for normality by the 1-Sample Kolmogorov - Smirnov Z test (K-S) . Thereafter, mean (±SE) trap yields on consecutive nights were compared using a 2- sample t test for data complying with normal distribution. Otherwise, the Mann Whitney rank sum test was applied. All statistical analyses were carried out on GraphPad PRISM®, version 5, (San Diego, CA).

## Results

### Timing the rotation of male genitalia

A total of 36 laboratory-reared (26°C) *Ph. sergenti* (F1) males were collected and examined at different times after eclosion. Males with fully rotated genitalia (Fig. 2D) were first observed amongst those collected 25 hours post-eclosion (Table 1).

**Table 1:** Timing the rotation of the external genitalia of male *Phlebotomus sergenti* reared in the insectary at 26°C.

Hours post-eclosion	Juvenile malesUn-rotated or partially rotated genitalia	Mature malesFully-rotated genitalia
0–14	6	0
0–20	7	0
0–25	10	3

### Baseline sand fly collection

In order to obtain baseline data on density and species composition of sand flies in different habitats, we sampled sand flies in and around four cave systems using CDC light traps with green light sticks (Fig. 1B). A total of 1,372 sand flies comprising 1,049 males and 323 females was trapped during six nights. The male sand flies were identified and shown to comprise 79% *Ph. sergenti* and >1% *Ph. papatasi*. The rest were *Sergentomyia* spp. The three cave systems where most flies were captured were selected for further study (marked 1, 2 & 3 in Fig. 1A).

In order to determine the presence of sand flies in and near artificial support walls, 50, A4 sticky traps were inserted horizontally into gaps between tiers of boulders and vertically between adjacent boulders of a support wall (Fig. 2F). Traps were collected the next day and 111 sand flies were removed from the sticky traps. Of these 75 were *Ph. sergenti* males all of which had fully rotated genitalia (Fig. 2D).

### Emergence traps

Rocky ledges near caves - Four tunnel emergence traps with sand fly-proof netting enclosing four large sticky traps (60×80 cm) and covering approximately 10–14 m^2^, were deployed for one night on lime-stone rock ledges above and below the caves (Fig. 1A asterisks). One female *Ph. sergenti* was captured in one of these traps. Fifteen *Ph. sergenti* (six males and nine females) were captured on a single large exposed sticky trap deployed in the same area.

### Rock mounds

Twelve sand flies, including 10 male *Ph. sergenti* were captured in emergence tunnel traps covering piles of rocks stacked upon rock ledges (Fig. 2B). The trap was deployed for one night in one location trapping no flies. Thereafter, the trap was moved to an adjacent location where three flies were trapped during the first night and nine male *Ph. sergenti* were trapped the following night. Six of these males had un-rotated or partially rotated genitalia (Fig. 2C), indicating they were emerging from a breeding habitat. Unfortunately, due to safety concerns, potential breeding sites in this loose-rock slope could not be investigated any further.

### Un-cracked soil/Dry Riverbeds

No sand flies at all were capture by a “tunnel” trap placed under an *Acacia* tree for three nights. During the same three nights, 127 sand flies (68 males) were trapped on four large sticky traps (12 trap/nights) placed next to the tunnel (Fig. 2A). Similarly, no flies were captured in an emergence “tunnel”-trap deployed over-night covering a small rock mound in the dry river bed. Thirteen emergence traps (Fig. 1F) were deployed for one night each over un-cracked soil in additional dry river beds and slopes around Kfar Adumim. No flies were captured in any of those.

### Caves

A total of 4,787 sand flies (*Phlebotomus* and *Sergentomyia*) were trapped inside and outside 3 covered caves over 18 nights ( = 185 trap/nights) using large sticky traps (Fig. 1D). Of these 3,468 (72%) were males, and the predominant species was *Ph. sergenti* accounting for 84% of all male sand flies. A significant proportion (25%) of the *Ph. sergenti* males had un-rotated or partially rotated genitalia suggesting proximity to breeding habitats (Table 2).

**Table 2:** Summary of sand fly catches using large sticky traps inside and outside caves covered by sand fly-proof netting.

	Inside caves	Outside caves	Total
Female ♀ sand flies	308	1,011	1,319
Male ♂ sand flies	1,247	2,221	3,468
Mature *Ph. sergenti* ♂♂	924	1,261	2,185
*Ph. sergenti* ♂♂ with un-rotated genitalia	197 (18%)	520(29%)	717(25%)

There were 85 trap/nights inside caves and 100 trap nights outside the caves.

**Figure 3:**
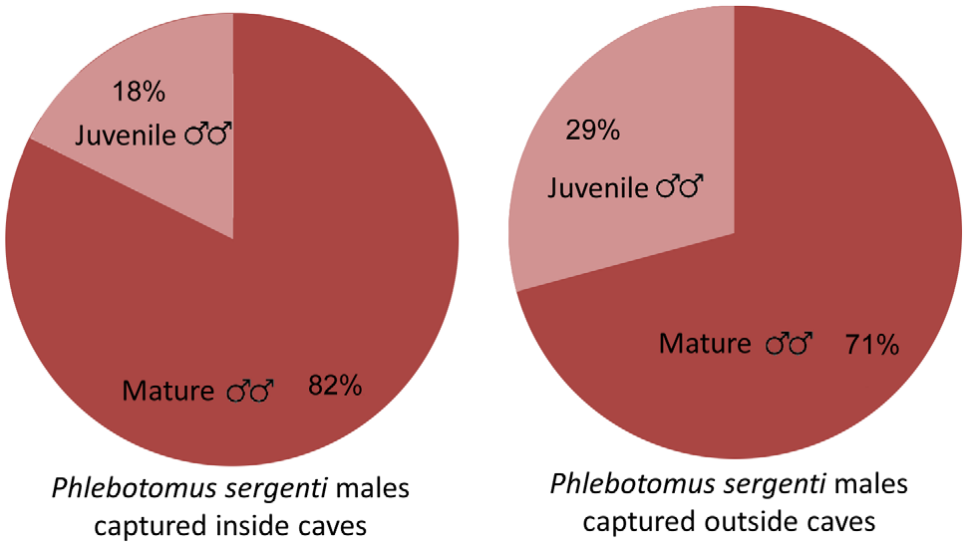
Percentage young males inside and outside caves. Percentage of juvenile ( = un-rotated genitalia) male *Phlebotomus sergenti* in caves (Left pie) and outside caves (Right pie). The difference was highly significant (χ ^2^ = 49.97, P<0.0001).

Sand flies trapped inside caves covered with sand fly-proof nets comprised 1,247 males, 90% of which were *Ph. sergenti*. A relatively high percentage (18%) of the *Ph. sergenti* males captured inside covered caves had un-rotated genitalia (Fig. 3).Of the sand flies trapped outside the caves, 2,221 were males and 80% of the male sand flies were *Ph. sergenti*. A significantly higher proportion of the *Ph. sergenti* males captured outside sealed caves had un-rotated genitalia (29%, χ ^2^ = 49.97, P<0.0001, Fig. 3). Thus, breeding sites were not limited to the sealed caves and sand flies were also emerging from neighboring caves, cracks, small holes or burrows (Table 2).

**Figure 4:**
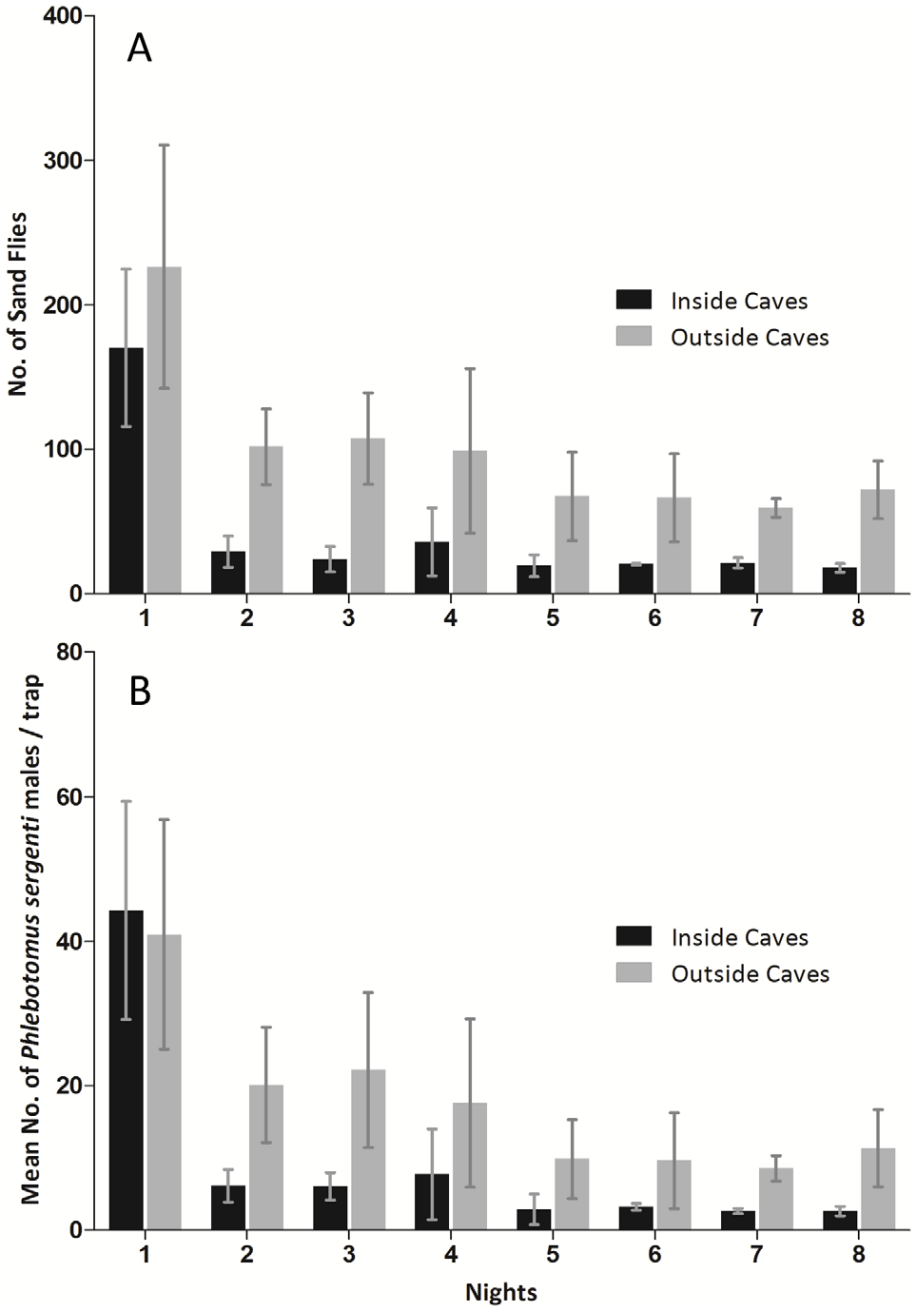
Sand fly catches inside and outside caves covered with sand fly-proof net. A) Mean number nightly (±SE) of sand flies captured on sticky traps inside and outside caves. The cave openings were covered with sand fly-proof nets. Sand flies were collected daily and the traps were smeared with fresh oil. The decline in sand fly numbers trapped inside caves observed during the second night of the caves' opening being covered, was statistically significant (two tailed t test, P = 0.0056). Although a parallel decline in numbers of sand flies outside the cave after the first night was also observed, it was not statistically significant (Mann – Whitney rank sum test, P = 0.4642). B) Mean number (±SE) of *Phlebotomus sergenti* males per sticky trap per night captured inside and outside caves. The decline in *Phlebotomus sergenti* males numbers trapped inside caves observed during the second night, was statistically significant (two tailed t test, P = 0.0019).

The number of sand flies captured during the first night inside covered caves was somewhat lower than those captured outside caves. Sand fly numbers dropped both inside and outside the caves on the second night of all experiments (Fig. 4A). The drop in numbers inside the caves was statistically significant (Two samplest test, P = 0.0056) while the decline in numbers of sand flies captured outside the caves was not statistically significant (Mann – Whitney rank sum test, P = 0.4642). After the second night, the numbers of sand flies remained more or less stable. An identical trend was observed amongst *Ph. sergenti* males which numbers inside sealed caves declined significantly after one night (Two samplest test, P = 0.0019).

### Support wall

In baseline collections 20 A4 sticky traps were inserted horizontally in gaps between boulders of the support wall. Sand flies were removed the next day and males were identified. Of the 111 sand flies, 75 were *Ph. sergenti* males, all of them with fully rotated genitalia (Fig. 2D).

To determine whether sand flies were breeding in the support wall, 50 A4 sticky traps were inserted in gaps between boulders along three, 7 m long sections of the wall. These sections were covered with sand fly-proof mesh. Additional 15 sticky paper traps were placed on stones and vegetation outside the mesh. The experiment lasted four nights and the sticky traps were collected and replaced every day. In all, 203 *Ph. sergenti* males were identified out of a total of 213 sand flies trapped during the experiment. Of the *Ph. sergenti* males 13% of those trapped inside and 19% of those trapped outside the net had un-rotated genitalia (Fig. 3, Table 3). The difference in the percentages of males less than 24 h old inside the netting and outside it were not significant (χ ^2^ = 1.402, P, ns).

**Table 3:** *Phlebotomus sergenti* males captured over 3 nights using A4 sticky traps that were placed in gaps between the boulders along a 10 m section of an artificial support wall (Fig. 1F - wall) and on rocks and vegetation (external).

	Inside Netting (wall)		Outside Netting (external)	
	Mature ♂♂	Juvenile ♂♂	Mature ♂♂	Juvenile ♂♂
Day 1	19	4(17%)	25	8(24%)
Day 2	6	2(33%)	9	1(10%)
Day 3	6	4(66%)	24	14(37%)
Day 4	37	0(0%)	43	1(2%)
Total	68	10(13%)	101	24(19%)

“Wall” traps were separated from “external” traps by a sand fly-proof netting.

### Soil analyses

The soil texture was predominantly sandy in eight of the ten sites sampled. Air drying of the soil samples for 72 hours resulted in insignificant reduction in gravimetric water content. Hygroscopic water content determined by heating for 24 h at 105°C varied between 2.09% to 6.26%. The highest values were found in caves and borrows and lowest ones outside caves and in the support wall. The pH values were uniformly slightly alkaline. Salinity calculated from the electric conductivity values was high in all samples. The highest values were measured in caves and the support wall - presumably due to these habitats being protected from rain. The organic matter content also varied widely with the higher values recorded in some of the caves and under an acacia tree (Table 3).

## Discussion

Caves and crevices as well as rodent burrows and cracked rocks have all been postulated to afford suitable environments for sand fly breeding [9]. However, no attempts were made to conclusively demonstrate that sand flies were in fact breeding in such habitats. In the current study we monitored adult activity as an indicator for sand fly resting and breeding sites. By sealing off caves with sand fly-proof netting, we were able to ascertain that sand flies captured inside were emerging from within the enclosed space. To separate possible resting populations from those emerging from pupae, we continued trapping inside sealed caves 2–7 additional nights. Although there was a significant decline in numbers of sand flies captured inside the cave after the first night, sand flies continued to be collected inside sealed caves over several nights (Fig. 4). If we assume that sand flies captured during the first night were mostly resting adults leaving their diurnal shelters to forage, the majority of flies captured during subsequent nights (2–8) can be considered as emerging from breeding sites [27].

Interestingly, in all five repetitions of the experiment in three different caves, sand fly numbers outside sealed caves also dropped after the first night, albeit insignificantly. Perhaps sand fly activity is restricted to a small area, close to their emergence site, where they use the same resting habitat night after night. In such a case, those sand flies trying to exit during the evening hours were stopped by the mesh and many were caught on the sticky traps. Similarly, sand flies attempting to enter the covered cave towards the end of the night were either captured on the external traps or eventually moved on to other suitable habitats nearby. These displaced sand flies were “lost” to the monitored cave's potential population during subsequent trapping nights. This scenario would explain the sharp decline in numbers observed on the second night both inside and outside the caves (Fig. 4).

The tendency of *Ph. sergenti*, the vector of zoonotic *L. tropica*, to congregate in and around their diurnal resting/breeding sites, which are frequently in rocky habitats with caves or boulder mounds inhabited by hyraxes, has been previously documented [21], [35]. In preceding studies performed in Kfar Adumim and elsewhere in Israel, it was shown that *Ph. sergenti* were abundant in caves and rocky slopes but conspicuously absent from nearby homes [19], [21], [36].

In our initial experiments we demonstrated that sand fly males with un-rotated genitalia can be considered young males that are active during the first night of adulthood (Table 1). Since such males were abundant inside sealed caves, these caves must have contained sand fly breeding habitats. However, since even higher percentages of young males were captured outside the covered caves (Figs. 3), it is clear that sand flies were also breeding in other sites not covered by nets. Our efforts to identify such places were largely unsuccessful and no flies were captured in emergence traps placed in various locations including rocky ledges close to the caves. One notable exception were young male sand flies with un-rotated genitalia captured using a tunnel-type emergence trap covering a pile of stones next to cave 1 (Fig. 1A marked with star). Hence, young males emerging from this pile (on nights when it was not covered by mesh) and neighboring caves and cracks, could have accounted for the ones captured on sticky traps outside covered caves. Although the topological conditions made it too dangerous to perform intensive studies in the rock pile, we do not believe sand flies were breeding in the pile itself since suitable larval habitats (organic matter, cool temperatures and high humidity) would not be expected in such a loose-rock pile. Thus, breeding probably took place in caves and caverns with openings under the rock pile. These may even have been contiguous with the large cave systems.

**Figure 5:**
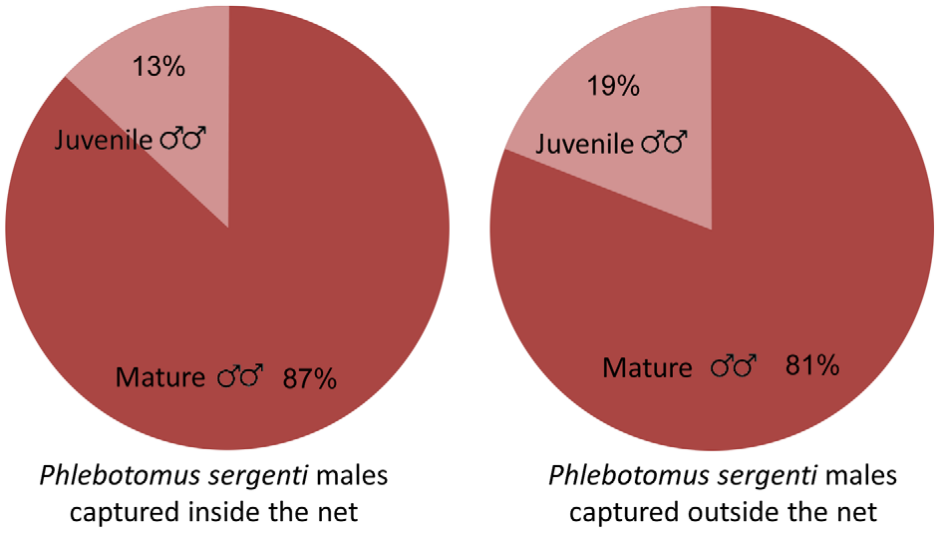
Percentage of young males in and near support walls. Percentage of juvenile ( = un-rotated genitalia) male *Phlebotomus sergenti* in the support wall (Left pie) and outside the support wall (Right pie). The differences were not statistically significant (χ ^2^ = 1.402, P, ns).

Male *Ph. sergenti* with un-rotated genitalia were also caught in and near an artificial support wall but in much smaller numbers than around caves (Table 3, Fig. 5). The presence of these young males indicates that sand fly breeding does take place within these walls. Although cracks are mostly too small for hyraxes, various rodents such as house mice (*Mus musculus*) and spiny mice (*Acomys cahirinus*) are plentiful in such walls (Warburg, unpublished). Young male *Ph. sergenti* captured outside the net probably emerged from the wall in adjacent areas not covered by the net or they may have flown from caves and burrows some 20 m downhill. The suitability of support walls constructed using large boulders leaving wide gaps for sand fly breeding, should be taken into consideration in future planning of residential neighborhoods.

No flies were captured in any of the emergence traps placed over bare soil, grass covered soil, dry river beds, valley slopes, rock-covered soil or dried sewage treatment basin. These negative findings indicate that sand flies emerge through visible cracks, burrows and cave openings and not from unbroken surfaces. We know that sand flies require habitats with stable temperatures and high humidity and such conditions would not be met at the upper horizons of desert soils. Moreover, the combustible organic matter in soils is not a suitable food source for sand fly larvae. Much like the rearing conditions used in insectaries, natural larval breeding habitats must contain composting animal feces and/or plant-derived matter as larval food [17].

The terrain where the current study was conducted was particularly difficult to study and there was no possibility of obtaining soil samples from the actual dwelling place of the larvae. Therefore, we extracted soil samples from productive caves, and compared them with samples taken from areas where sand flies do not breed. All the samples were rather desiccated and characterized by high salinity. The organic matter content was rather low but somewhat higher inside caves and under a particular tree. On the whole we cannot deduce too much from these results as differences between productive areas and barren ones were inconsistent (Table 4). Caves did contain ample quantities of rock hyrax feces. The fecal pellets found close to the opening of the cave were hard and dry. However, deeper inside caves pellets would be expected to be more humid and, therefore, softer making them suitable as sand fly larval food. Although the soil analyses do not pertain to the exact location where larvae dwell, they were included in this report as putatively important points of reference for future studies (by us and others).

**Table 4:** Summary of soil parameters in several sand fly resting/breeding and in control habitats in Kfar Adumim.

Sample	Soil texture	Hygroscopic Water content (%)	pH	Electrical conductivity (dS 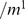 )	Salinity (g/l)	Organic matter (%)
Cave 1 Lobby	Sandy Clay Loam	5.15	7.82	13.53	8.66	14
Outside Cave 1	Sandy Clay Loam	2.56	8.11	0.775	0.49	3
Burrow near cave 1	Sandy Clay Loam	6.26	7.5	13.14	8.41	41*
Cave 2 Lobby	Sandy Clay Loam	4.38	7.34	17.87	11.44	14
Cave 3 Tunnel	Sandy Loam	4.33	7.58	5.67	3.63	25
Outside Cave 3	Sandy Loam	2.93	7.65	0.953	0.61	12
Burrow entrance	Clay Loam	4.11	7.47	13.37	8.56	11
Under acacia tree	Sandy Loam	5.15	7.61	5.65	3.61	20
Crack on cliff face	Sandy Loam	2.09	7.22	21.8	13.95	6
Gap in artificial support wall	Clay Loam	2.40	8.07	1.32	0.84	2

*: **Contained visible rodent fecal pellets.**

Our results are in accord with previous studies that postulated the existence of larval breeding habitats in rocky slopes, caves and support walls in Kfar Adumim, based on the high proportion of male sand flies captured near such habitats [21]. Interestingly, other studies performed in the Judean Desert suggested sand fly breeding and resting occurs primarily in valley floors covered with vegetation [27]. Our efforts failed to capture any sand flies emerging from soil in valley floors or slopes with or without vegetation or stones. These differing findings may be due to the fact that Muller et al [27] were dealing primarily with *Ph tobbi* and *Ph major* while our study and that of Orshan et al [21] focused on *Ph. sergenti*.
